# Construction and application of SARS-CoV-2 protein ontology (CoVPO)

**DOI:** 10.1371/journal.pone.0317589

**Published:** 2025-05-12

**Authors:** Aaron Nkhata, Xiaona Shi, Yongjuan Zhang, Heng Chen

**Affiliations:** 1 Institute of Big Data and Information Technology, Wenzhou University, Wenzhou, China; 2 Key Laboratory of Intelligent Informatics for Safety & Emergency of Zhejiang Province, Wenzhou University, Wenzhou, China; 3 Shanghai Institute of Nutrition and Health, Chinese Academy of Sciences, Shanghai, China; West Pomeranian University of Technology, POLAND

## Abstract

The emergence of the SARS-CoV-2 virus and the resulting COVID-19 pandemic brought forth an urgent need for an in-depth molecular understanding, organization, and data integration to expedite therapeutic and preventive strategies. An essential approach to achieving this is through the development of a well-structured ontology of SARS-CoV-2 proteins. In response, this paper introduces CoVPO, a SARS-CoV-2 protein ontology that improves upon existing ontologies on protein function annotation and viral-to-viral protein interactions, highlighting their limited scope in covering all aspects of SARS-CoV-2 proteins. CoVPO extends classes from other relevant ontologies. Terms, annotations, and synonyms are added with proper definitions, clear origins, and an interaction map of viral-to-viral protein interactions is captured. We demonstrate CoVPO’s application in an information retrieval system, expanding user queries by adding related terms or concepts. This approach helps overcome issues like term mismatch and improves the retrieval of relevant documents. The feasibility and superiority of the domain ontology model are demonstrated through experiments, showing that it outperforms traditional keyword-based searches and provides grounds for further research and discussion in the SARS-CoV-2 protein domain.

## 1. Introduction

The outbreak of the SARS-CoV-2 (Severe acute respiratory syndrome coronavirus 2) virus and its subsequent global impact in the form of the COVID-19 (coronavirus disease 2019) pandemic not only did it pose unprecedented challenges to public health but also underscored the critical importance of comprehensively understanding the intricacies of this novel coronavirus [1]. As scientists and researchers across the globe race against time to develop effective therapeutic and preventive strategies, the significance of a well-structured ontology for SARS-CoV-2 proteins becomes increasingly apparent.

This paper addresses the urgent need for a systematic approach to organizing and integrating data related to the SARS-CoV-2 proteins domain [2]. In the wake of a pandemic that disrupted lives and societies, our objective is to enhance our collective understanding of the virus, offering a more efficient and informed pathway towards combating its effects. The construction of a SARS-CoV-2 protein ontology serves as a pivotal step in this pursuit, building upon foundational ontologies while extending the entities to encompass a broader and more detailed framework.

The rationale behind this endeavor lies in the realization that comprehensive insights into SARS-CoV-2 proteins are essential for developing novel therapies, vaccines, and diagnostic tools. Structural proteins, such as spike (S) protein, envelope (E) protein, and membrane (M) protein, play a crucial role in the virus’s life cycle [3], while non-structural proteins, such as non-structural protein 5 (nsp5) and non-structural protein 12 (nsp12), are essential for viral replication and transcription [4]. Accessory proteins, including ORF3a, ORF6, and ORF7a, play various roles in the virus’s pathogenesis, such as immune evasion and modulation of host cell signaling pathways [5]. Researchers are currently discussing targeting these proteins as a potential therapeutic approach. This highlights the importance of understanding the role of each protein in the virus’s pathogenesis and how targeting them can provide potential therapeutic options for treating COVID-19. Therefore, by creating a domain-specific ontology that enriches the existing related ontologies, such as ID0-COVID-19 [6] with additional terms, annotations, synonyms, and an interaction map of the virus’s proteins, we aim to facilitate not only a more organized knowledge base but also foster enhanced collaborations and data sharing within the scientific community.

Furthermore, this ontology is designed to capture and represent the predominant essential entities and relevant concepts within the SARS-CoV-2 proteins research domain. It aims to capture viral-to-viral protein interactions and provide a detailed account of the methodology and design principles behind the SARS-CoV-2 protein ontology, as well as its practical application in an information retrieval system.

In this paper, we systematically examined various existing ontologies, focusing on specific topics such as protein function annotation and viral-to-viral protein interaction. Our analysis revealed that these ontologies exhibit a limited scope, failing to comprehensively cover all aspects of SARS-CoV-2 proteins. Notably, there is currently no comprehensive model addressing the entirety of SARS-CoV-2 proteins and their viral-to-viral interactions. Existing literature indicates the presence of ontologies designed to capture knowledge related to SARS-CoV-2, albeit with differing focuses and scopes. Recognizing this gap, our work is motivated by the need to address and fill this existing void. The main contributions of this paper include:

(1) To cover every aspect of SARS-CoV-2 viral-to-viral protein interaction.
(2) To develop a schema called CoVPO as a global data model to annotate SARS-CoV-2 protein information.
(3) To include detailed terms, annotations, synonyms, and clear definitions of SARS-CoV-2 protein within CoVPO, enhancing data integration and retrieval.
(4) Through experiments, the paper shows that the domain ontology model (CoVPO) outperforms traditional keyword-based searches. This highlights the feasibility and advantages of using ontologies for information retrieval in the SARS-CoV-2 protein domain.

We hope this work will be a valuable resource for researchers, clinicians, and policymakers, aiding their efforts to combat COVID-19 and prepare for future viral threats by offering a deeper understanding of the proteins crucial to the pathogenicity and biology of SARS-CoV-2.

## 2. Related work

Ontologies play a crucial role in the integration of data, information, and knowledge, as well as in the realm of big data analysis, particularly prevalent in biomedical data. Dealing with a global pandemic is a knowledge-intensive process. As a result, several ontologies have been developed related to the COVID-19 pandemic. In this section, we describe the related work that we explored before developing the SARS-CoV-2 protein ontology.

Building on these foundational efforts, The Gene Ontology (GO) [7] provides a structured, precisely deﬁned, common, and controlled vocabulary for describing the roles of genes and gene products in any organism. In the context of the COVID-19 pandemic, the application of GO has emerged as a crucial tool in deciphering the complex interactions between the SARS-CoV-2 virus and the host organism. GO provides a structured framework for annotating and categorizing the diverse array of genetic and functional data associated with COVID-19.

Similarly, The Coronavirus infectious Disease Ontology (CIDO) [8] offers a structured vocabulary that aims to represent knowledge related to coronavirus infectious diseases. CIDO is focused on multiple areas of COVID-19, including transmission, etiology, epidemiology, pathogenesis, diagnosis, treatment and prevention.

Furthermore, The COVID-19 ontology [9] delineates key entities associated with the novel coronavirus (SARS-CoV-2), with a particular focus on chemical entities relevant to drug repurposing. This ontology encompasses 2,270 classes of concepts and 38,987 axioms, comprising 2,622 logical axioms and 2,434 declaration axioms. It elucidates the functions of molecular and cellular entities in virus-host interactions and the virus life cycle. Additionally, it encompasses a broad range of medical and epidemiological concepts interconnected with COVID-19.

In addition, The COVID-19 surveillance Ontology (COVID-19) [10] depicts primary care, public health, virology, clinical research, and clinical informatics from multiple medical records systems. The ontology is constructed as a taxonomy with only 32 classes.

Complementing these efforts, The Ontology for Collection and Analysis of Covid-19 Data (CODO) [11] provides a framework for gathering and examining data related to the COVID-19 pandemic. This ontology presents a standards-based open-source model that incorporates diverse data from various sources. Its development involved the analysis of multiple COVID-19 data sources, encompassing datasets, literature, and services.

Moreover, The Coronavirus Disease Ontology (CovidO) [12] groups multiple existing ontologies to build a global data model. The ontology has 175 classes, 165 properties, 4,141 triples, and 645 individuals with 264 nodes and 308 edges. The ontology consolidates and extends COVID-19 case information, disease-symptoms-treatment, COVID-19 impact, research, news related to COVID-19 and resources from different ontologies.

Among the ontologies discussed here, the COVID-19 ontology [9] and the Coronavirus Infectious Disease Ontology (CIDO) [8] are the most relevant. However, these ontologies have a limited scope and do not comprehensively cover all aspects of SARS-CoV-2 proteins, including viral-to-viral protein interactions and other facets.

## 3. Methodology

**Fig 1:**
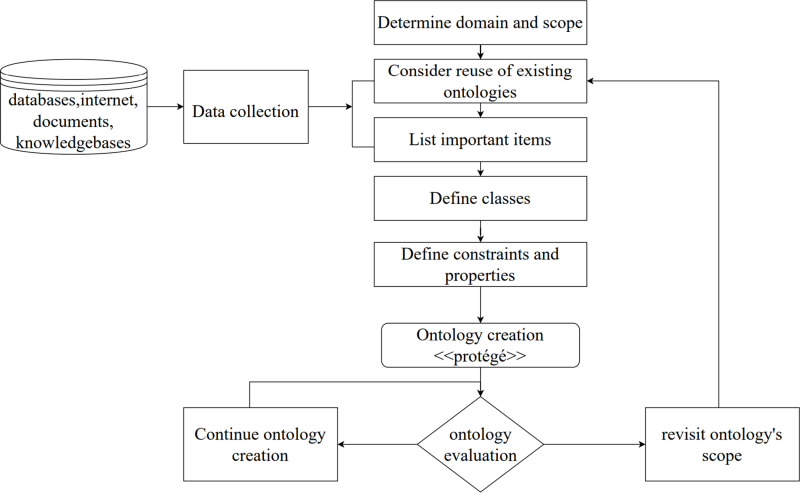
CoVPO development process based on Stanford seven-step method.

**Fig 2:**
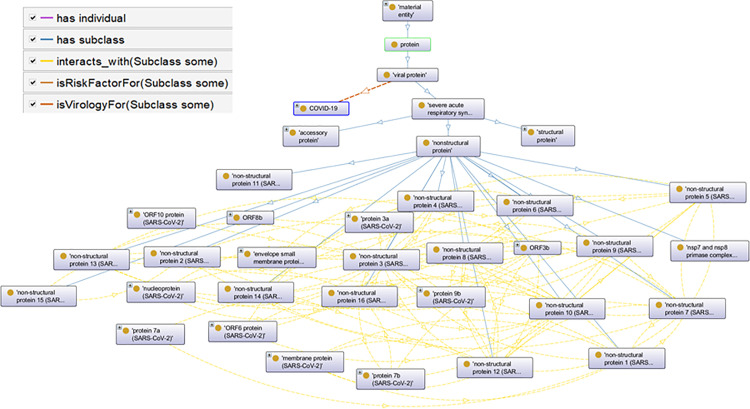
Partial overview of the ontology’s inheritance relationships and object properties.

This section outlines the methodology employed in the design and development of the CoVPO ontology. Various methodologies for designing ontologies exist in the literature, and among the cutting-edge approaches are the Stanford Seven-Step Method [13], Skeletal Methodology [14], TOVE Method [15], Methontology Method [16], etc. CoVPO’s design methodology is predominantly shaped by the Stanford Seven-Step Method, a systematic approach to constructing a domain ontology. The Stanford Seven-Step methodology outlines a series of guiding principles for ontology design, as illustrated in Fig 1 and elaborated in Fig 2.

**S1: Define scope** – this step involves identifying the specific subject area and deciding what aspects of that domain will be covered by the ontology. As discussed above CoVPO’s main purpose is to provide comprehensive insights into SARS-CoV-2 proteins and to be used to develop semantic services and applications. Also, to enable researchers, data publishers, hospitals and other various organizations to annotate and describe SARS-CoV-2 protein information.

**S2: Consider reuse** – reusing existing ontologies is a prudent approach to avoid reinventing the wheel. It promotes interoperability, consistency, and efficiency in information systems. It fosters standardization, facilitates knowledge integration, and encourages community collaboration. It is required to develop and reuse domain vocabularies. According to [17], the concept of Ontology reuse can be viewed in two ways: (1) the process involves assembling, extending, specializing, and adapting other ontologies, which become integral components of the resulting ontology; or (2) the act of merging distinct ontologies with a common subject into a single ontology that unifies all of them. The former, referred to as integration, is the approach taken in the development of CoVPO.

**S3: Listing important terms** – to enumerate important terms for the ontology, we extracted various aspects of the virus’s components from data repositories (SWISS-MODEL, UniProtKB, Protein Data Bank, NCBI Virus etc.), newspaper articles and literature from various databases. Some of the significant extracted terms are: SARS-CoV-2 protein, viral-to-viral protein interactions, Structural Proteins, Non-Structural Proteins (nsps), Accessory Proteins, Protein function etc.

**S4: Define classes** – this step involves organizing and structuring the knowledge gathered from previous steps into respective classes. These classes represent concepts or entities within the domain. The relationships between these classes are organized into a hierarchical structure, allowing for the classification and categorization of knowledge.

**S5–S6: Define properties and constraints** – to accurately represent the knowledge, classes need to be associated with properties. These properties describe various attributes and relationships between classes. In this step, we also specify the characteristics of these properties (intrinsic, symmetric, etc.), including data types and other constraints, to ensure that the ontology is both comprehensive and well-structured.

**S7: Create instances** – instances are specific examples of classes within the ontology. Instances provide concrete data points that can be used in real-world applications. Instances also bring the ontology to life, making it practical for data integration, reasoning, and information retrieval.

**S8: Evaluation** – this stage involves verifying how well the ontology has satisfied the requirements. It gauges the technical competence of the ontology. A reasoner is used to check the syntactic structure and the consistence of the ontology. The domain knowledge and knowledge structure are manually evaluated by domain experts. Currently, there is no easy and automatic way to achieve the latter.

## 4. Properties and classes of the CoVPO ontology

**Fig 3:**
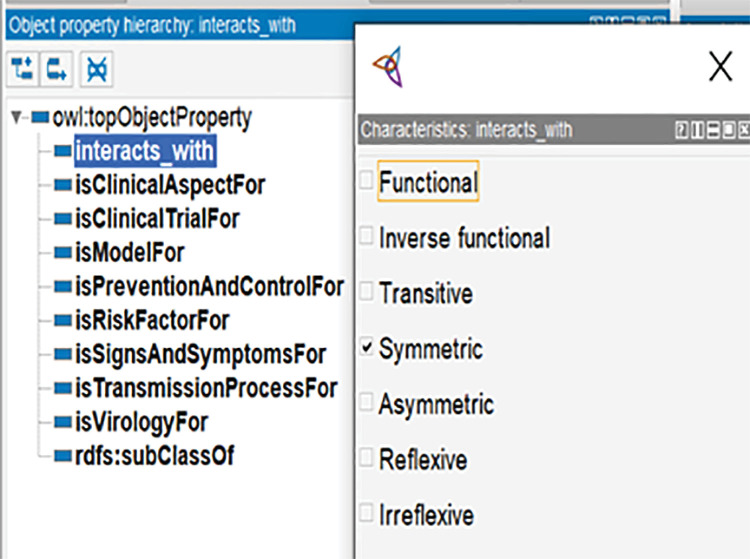
CoVPO object properties and their characteristic.

In OWL (Web Ontology Language), a language designed to represent rich and complex knowledge about things, groups of things, and the relationships between things, properties are used to describe relationships between individuals or to specify attributes of individuals. There are two main types of properties in OWL: object properties and data properties. Object properties can be further classified into sub-properties, such as symmetric, transitive, functional, and inverse functional properties, depending on their characteristics. A symmetric property is one where if the tuple < x, y>  is in the property then the tuple < y, x > must also be in the property. An example of a symmetric property in CoVPO is interacts_with as shown in Fig 3. This means that if non-structural protein 8(nsp8) interacts with non-structural protein 7 (nsp7) then the reasoner automatically infers that nsp7 interacts with nsp8.

**Fig 4:**
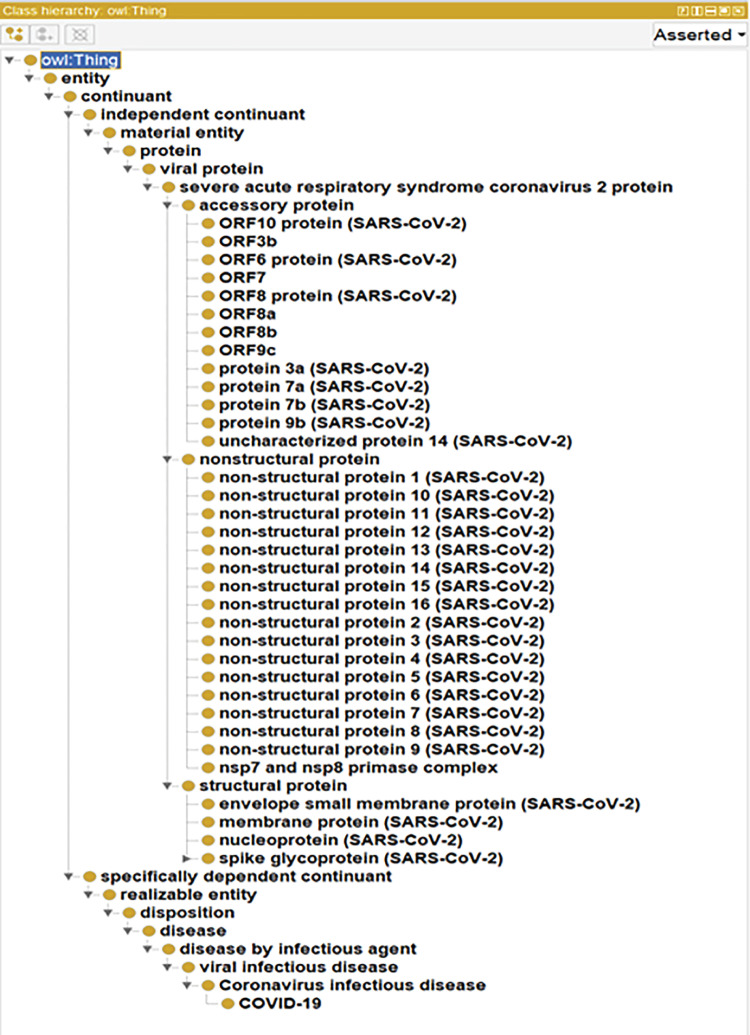
Hierarchy of SARS-CoV-2 proteins in CoVPO ontology.

The classes shown in Fig 4 represent concepts or entities within the SARS-CoV-2 protein domain. The relationships between these classes are organized into a hierarchical structure, allowing for the classification and categorization of knowledge. Hierarchical or non-hierarchical relationships can exist between terms. Non-hierarchical relationships are formed by specifying object attributes, which include both semantic and data characteristics. Semantic attributes explain the connection between terms, while data attributes represent links between terms and non-terms. Hence, when enumerating or listing important terms of the ontology, it is essential to consider the relationships between these terms. The hierarchical relationship between each term is confirmed according to Protein Ontology (PRO), and the non-hierarchical relationship between each protein and related terms are established according to the literature studies, UniProt, and PRO.

## 5. CoVPO evaluation and it’s application

In this section, we describe two methods we used to evaluate the CoVPO ontology. It is evaluated using SPARQL queries and an information retrieval system. The performance of the system is measured using recall, F1 Measure, and precision metric.

### 5.1. SPARQL query evaluation

SPARQL is a query language used to retrieve and manipulate data stored in Resource Description Framework (RDF) format. The significance of SPARQL queries lies in their ability to access and manipulate RDF data, which is fundamental to the semantic web. SPARQL queries enable users to search, filter, and aggregate RDF data, facilitating data integration and interoperability across diverse sources. As the volume and complexity of linked data continue to grow, the importance of SPARQL as a query language becomes increasingly pronounced. The basic structure of a SPARQL query consists of a SELECT clause, WHERE clause, and optional modifiers such as ORDER BY, GROUP BY, and LIMIT. Like SQL, it can also delete, transform, and insert data [18].

**Fig 5:**
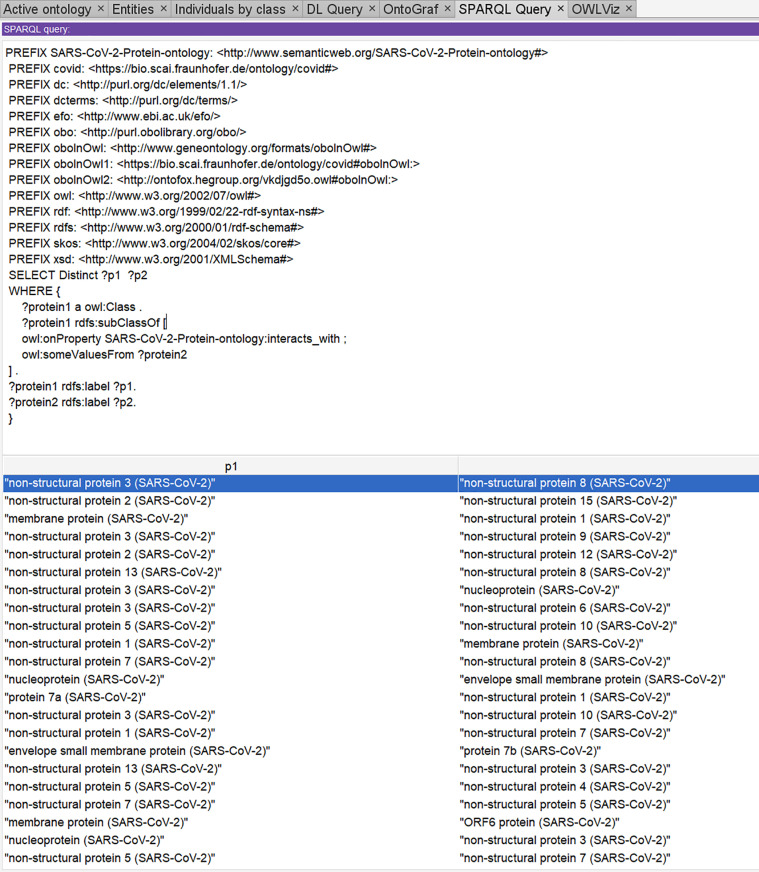
Sparql query to extract viral-to-viral protein interaction.

**Fig 6:**
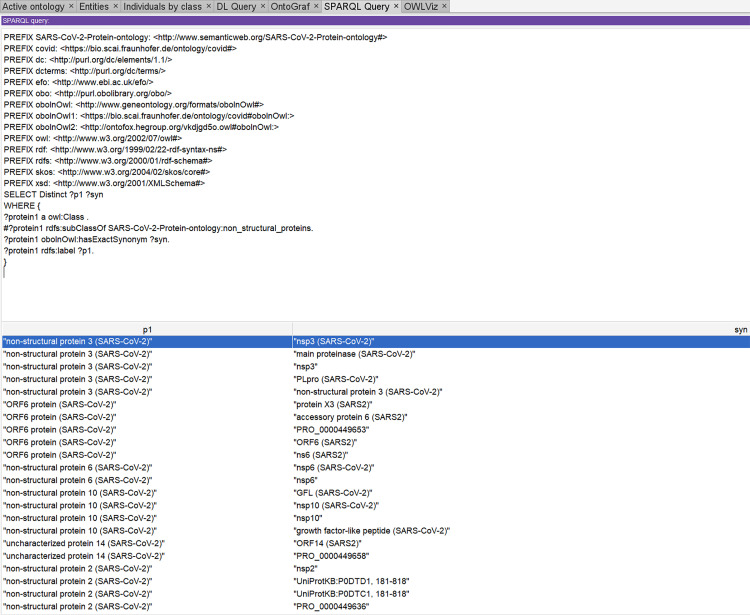
Sparql query to extract the entity’s exact synonyms.

Figs 5 and 6 display SPARQL queries using the Snip SPARQL query plugin in protégé. The SPARQL syntax shown in Fig 5 is for the query *“list all the viral-to-viral protein interactions of SARS-CoV-2”* and the SPARQL syntax shown in Fig 6 is for the query “*lists all the entity’s exact synonyms of non-structural proteins*”

### 5.2. Informational retrieval system

Information retrieval is the process of finding relevant documents within large datasets. With the increasing amount of data and the growing need for high-quality search results, traditional retrieval methods often fail to provide the best outcomes. Ontology, a system for organizing knowledge, is essential for improving the efficiency and effectiveness of information retrieval in knowledge management. Ontologies can be used to find the most related words for the query, by measuring the similarity between the query words that are semantically related words based on domain or global knowledge a detailed research work is presented by [19].

Research on the application of ontology in information retrieval has garnered significant attention in recent years, resulting in notable advancements. Researchers are exploring innovative ways to leverage ontologies to improve search accuracy, enhance recommendation systems, and develop more intuitive knowledge graphs. These advancements aim to address existing challenges in information retrieval, such as vocabulary mismatch [20], and provide users with a more efficient, effective, and personalized search experience.

One area where ontology has shown promising results in information retrieval is in improving user search query and accuracy [21]. By incorporating ontologies in information retrieval systems, search engines can better understand the semantics and context behind user queries, leading to more precise and relevant search results. In addition, the incorporation of ontology in recommendation systems allows for a deeper understanding of user preferences and interests [22]. This enables the system to provide more personalized and accurate recommendations, enhancing the overall user experience. Another application of ontology in information retrieval is the development of knowledge graphs. These knowledge graphs serve as interconnected networks of information, where concepts and entities are linked based on their semantic relationships. This not only helps in organizing and structuring information but also provides a comprehensive view of the knowledge domain, allowing users to explore related concepts and discover new insights.

Moreover, the use of ontology in information retrieval systems helps bridge the gap between user queries and the underlying database structure. Traditionally, users are expected to have a deep understanding of the database structure and write complex search requests to retrieve information accurately. With the integration of ontology, users can express their information needs in a more natural and intuitive manner, allowing the system to interpret the query and retrieve relevant information based on the semantic relationships defined in the ontology. Therefore, our main focus in this section is to show the application of CoVPO in improving the information retrieval process on text documents by means of semantic query expansion. Targeting the search onto SARS-CoV-2 protein domain and expanding the search query to be relevant to this specific domain.

### 5.3. System architecture

**Fig 7:**
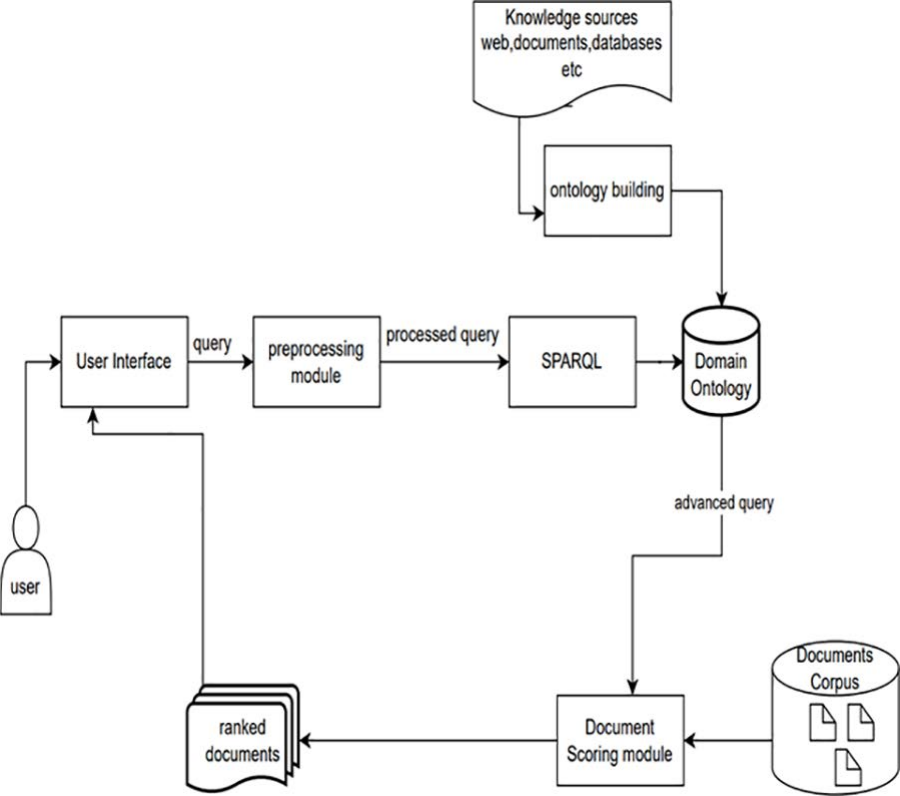
CoVPO information retrieval system architecture.

The system architecture of the CoVPO-based information retrieval is depicted in Fig 7. When a user accesses the user interface, they input search terms and select a search type, then submit the form. The Flask server processes this request, extracting and splitting the search terms into keywords, stemming, and removing stop words using natural language toolkit (NLTK). If the search type is ontology-based, SPARQL queries are executed on an RDF knowledge graph to find related terms, which are added to the keywords. The application reads text files from the document corpus and calculates term frequencies (TF) and document frequencies (DF) to compute TF-IDF vectors for each document. The system then uses cosine similarity to compare these TF-IDF vectors with the query vector, ranking documents based on their similarity scores, with higher scores indicating greater relevance to the query. The ranked list of documents is sent back to the client and displayed on the results page.

### 5.4. Measures used

#### 5.4.1. Recall.

Recall measures the completeness of the retrieval process. It calculates the proportion of relevant documents retrieved by the system out of all the relevant documents available for a given query. High recall indicates that the system retrieves most of the relevant documents, minimizing the chance of missing relevant information. However, high recall does not guarantee high precision, as the system may also retrieve many irrelevant documents. The formula for recall is given as follows:


$$Recall=\frac{numberofrelevantdocumentsretried}{Totalnumberofrelevantdocuments}$$

$$=\frac{truepositive}{truepositive+falsepositive}.$$ (1)

#### 5.4.2. Precision.

Precision measures the accuracy of the retrieved documents. It calculates the proportion of relevant documents among all the documents retrieved by the system for a given query. High precision indicates that the system retrieves mosy relevant documents, minimizing irrelevant ones. However, high precision does not guarantee high recall, as the system may miss some relevant documents. The formula for precision is given as follows:


$$Precision=\frac{numberofrelevantdocumentsretried}{Totalnumberofdocumentsretrieved}$$

$$=\frac{truepositive}{truepositive+falsepositive}$$ (2)

#### 5.4.3. F1 measure.

F1 measure combines precision and recall into a single metric to provide a balanced evaluation of the system’s performance. It is the harmonic mean of precision and recall, ensuring that both metrics are taken into account. The F1 measure is particularly useful when there is an uneven class distribution between relevant and irrelevant documents. The formula for F1 Measure is given as follows:


$$F1=\;\frac{2\times Precision\times Recall}{Precision+Recall}$$ (3)

### 5.5. CoVPO use case evaluation

In this section, CoVPO is evaluated using a prototype information retrieval system by leveraging query expansion technique (QE). QE is a technique used in information retrieval and search engines to improve search results by enhancing the original query with additional terms. The goal is to better match the user’s intent and retrieve more relevant documents.

Several semantic query expansion (QE) approaches are available to address the vocabulary mismatch problem, including linguistic, ontology-based, and mixed-mode methods [23]. The primary benefit of using semantic query expansion (QE) with an ontology domain is that the structured knowledge is consistently integrated into the expansion process, providing clear context for the query. This approach was adopted in the evaluation of CoVPO to enhance unclear queries. In this method of semantic QE, the query term is expanded with related terms from the ontology. The expanded terms are then processed by the information retrieval system to achieve more accurate results.

**Fig 8:**
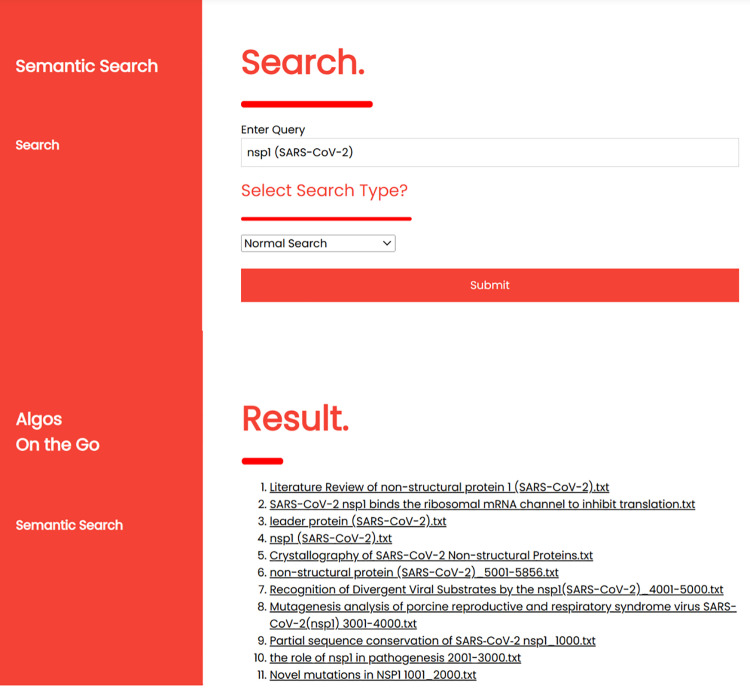
Traditional keyword-based retrieval method.

**Fig 9:**
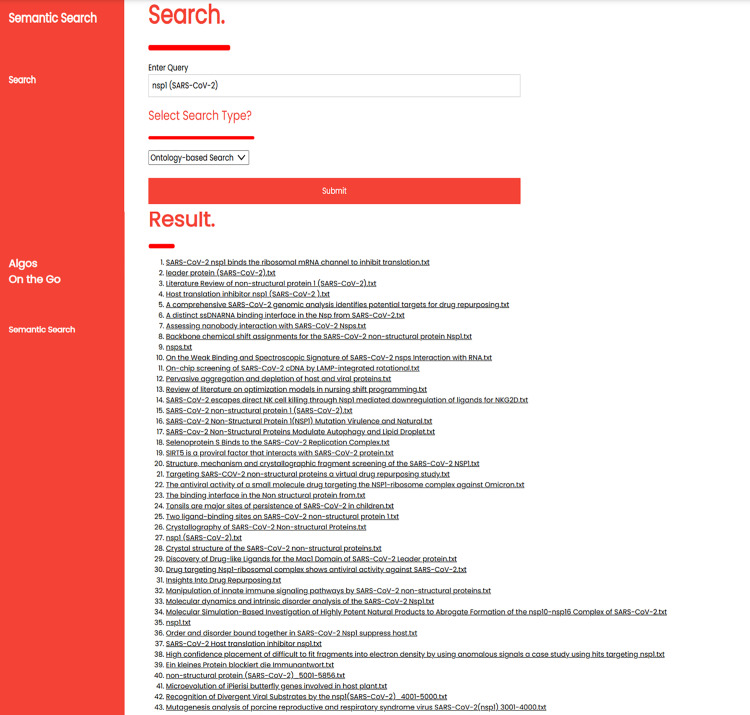
CoVPO-based retrieval method.

When conducting CoVPO evaluation using an information retrieval system, predefined queries known as test topics are formulated to define information needs. Additionally, selection of relevant documents corresponding to each query are identified. This step forms the basis for comparing the effectiveness of CoVPO-based and traditional keyword-based information retrieval system in fulfilling the retrieval requirements of a given set of predefined queries, as shown in Figs 8 and 9. By assessing how well the systems perform in recall ratio, precision and F1 Measure for these predefined queries, the effectiveness of CoVPO is evaluated.

#### 5.5.1. Tradition-keyword based query.

Before performing the query shown in Fig 8, a set of ground-truth is established. This includes determining the correct number of documents that should be retrieved by the system when the user selects the normal search (traditional-keyword) option. After running the query, a system performance analysis using the F1 measure evaluation is performed. Below is a query based on the keyword “nsp1 (SARS-CoV-2)” performed under the normal search option. The query retrieves 11 documents out of the 45 expected documents that are relevant to the query “nsp1 (SARS-CoV-2)”

Based on the query results shown above, the recall, precision is calculated, and the F1 score is as follows:


$$F1=\frac{2\times 1\times 0.2444}{1+0.2444}=0.3927=39.27\%$$ (4)

A lower F1 score of 39.27% suggests that there is an imbalance between precision and recall. The system is achieving high precision of 100% but at the expense of lower recall. This imbalance affects the overall effectiveness of the retrieval system in meeting users’ information needs. Furthermore, an F1 score of 39.27% indicates that while the system may be performing adequately, there is still room for enhancement to achieve a better balance between precision and recall, thereby improving its overall effectiveness in information retrieval tasks.

#### 5.5.2. CoVPO-based expansion query.

CoVPO-based query expansion retrieval automatically enhances the original search query by adding relevant and meaningful terms. Fig 9, shows a user query based on the keyword “nsp1 (SARS-CoV-2).”

Based on the query results shown above, the recall and precision are calculated, and the F1 score is as follows:


$$F1=\frac{2\times 1\times 0.9555}{1+0.9555}=0.9772=97.72\%$$ (5)

An F1 score of 97.72% suggests that the system achieves a high overall effectiveness in retrieving relevant documents while minimizing irrelevant documents. The high percentage rate indicates an excellent balance between precision and recall, with a slight emphasis on precision due to the precision being 100%. A recall of 95% indicates that the system has successfully retrieved 95% of all relevant documents, indicating a high coverage of relevant information retrieved. This suggests that the system is highly accurate and reliable in retrieving relevant information for the given query. Therefore, CoVPO-based expansion query enhances the retrieval of user information needs in the SARS-CoV-2 protein domain.

#### 5.5.3. CoVPO vs keyword retrieval on various datasets.

**Fig 10:**
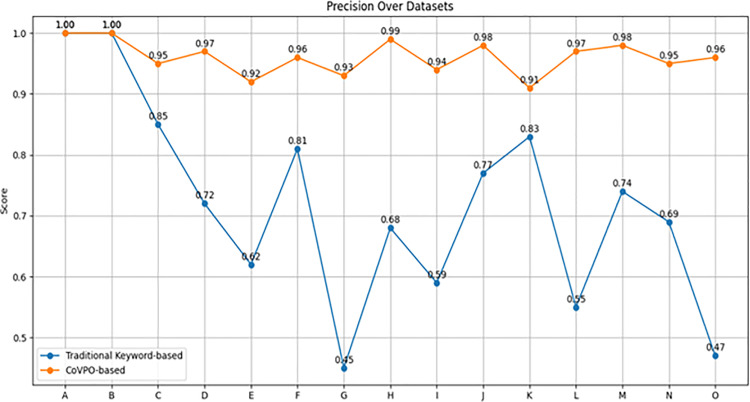
Precision comparison across datasets.

**Fig 11:**
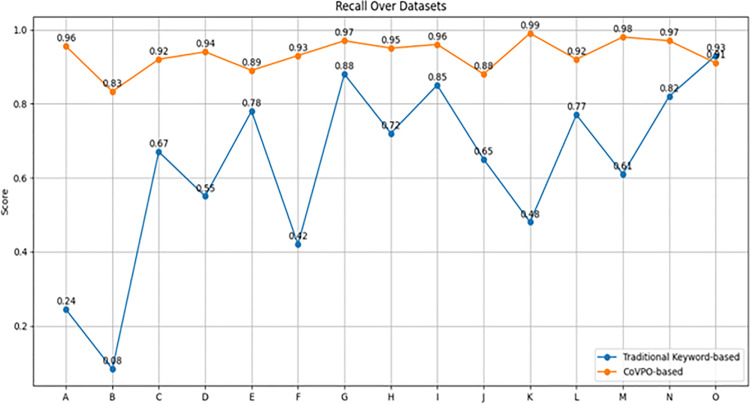
Recall comparison across datasets.

Table 1 shows the Precision, Recall, and F1 Score over the different datasets for both Traditional Keyword-based and CoVPO-based methods. Each metric is plotted separately to clearly show the performance trends across datasets. See Figs 9–11.

**Table 1:** Comparison of keyword and CoVPO-based retrieval method across diverse datasets.

Dataset	Method	Precision	Recall	F1 Score
Dataset A	Traditional Keyword-based	1	0.2444	0.3927
	CoVPO-based	1	0.9555	0.9772
Dataset B	Traditional Keyword-based	1	0.0833	0.154
	CoVPO-based	1	0.833	0.909
Dataset C	Traditional Keyword-based	0.85	0.67	0.75
	CoVPO-based	0.95	0.92	0.94
Dataset D	Traditional Keyword-based	0.72	0.55	0.62
	CoVPO-based	0.97	0.94	0.96
Dataset E	Traditional Keyword-based	0.62	0.78	0.69
	CoVPO-based	0.92	0.89	0.91
Dataset F	Traditional Keyword-based	0.81	0.42	0.55
	CoVPO-based	0.96	0.93	0.95
Dataset G	Traditional Keyword-based	0.45	0.88	0.59
	CoVPO-based	0.93	0.97	0.95
Dataset H	Traditional Keyword-based	0.68	0.72	0.69
	CoVPO-based	0.99	0.95	0.97
Dataset I	Traditional Keyword-based	0.59	0.85	0.69
	CoVPO-based	0.94	0.96	0.95
Dataset J	Traditional Keyword-based	0.77	0.65	0.69
	CoVPO-based	0.98	0.88	0.93
Dataset K	Traditional Keyword-based	0.83	0.48	0.61
	CoVPO-based	0.91	0.99	0.95
Dataset L	Traditional Keyword-based	0.55	0.77	0.64
	CoVPO-based	0.97	0.92	0.95
Dataset M	Traditional Keyword-based	0.74	0.61	0.67
	CoVPO-based	0.98	0.98	0.98
Dataset N	Traditional Keyword-based	0.69	0.82	0.75
	CoVPO-based	0.95	0.97	0.96
Dataset O	Traditional Keyword-based	0.47	0.93	0.63
	CoVPO-based	0.96	0.91	0.94

Fig 10 shows that CoVPO-based retrieval method maintains high precision across almost all datasets, consistently scoring close to or at 1.0. This indicates that the CoVPO-based method is highly accurate in identifying relevant items. In contrast, the Traditional Keyword-based Method shows more variability in precision. While it achieves perfect precision in several datasets (e.g., A, B), it drops significantly in others (e.g., E, F,G). These datasets include terms with synonyms and ambiguous meanings, which the keyword-based method struggles to handle effectively. This comparison underscores the robustness of the CoVPO-based method in managing complex datasets where ambiguity and synonymy are present.

Fig 11 shows that CoVPO-based Method consistently maintains high recall across most datasets, indicating it effectively identifies nearly all relevant items. In contrast, the Traditional Keyword-based Method’s recall fluctuates significantly, with very low values in some datasets (e.g., A, B, F) and higher values in others. This suggests it misses many relevant items in certain datasets. where ambiguity and synonymy are present.

**Fig 12:**
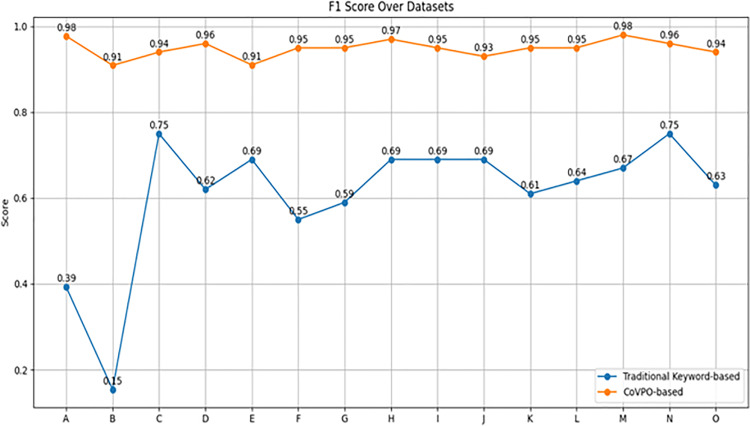
F1 Score comparison across datasets.

Fig 12 shows that CoVPO-based method maintains a high F1 Score across all datasets, reflecting a good balance between precision and recall. In contrast, the Traditional Keyword-based method’s F1 score varies widely, reflecting inconsistencies in precision and recall. It is notably lower in datasets where either precision or recall is very low. (e.g., B).

## 6. Conclusion

This work introduces the SARS-CoV-2 protein ontology, known as the Coronavirus Protein Ontology (CoVPO). It systematically examines various existing ontologies and extends relevant classes from them. The ontology includes well-defined terms, annotations, synonyms, and their clear origins, as well as a comprehensive viral-to-viral protein interaction map. Additionally, the effectiveness of this ontology is demonstrated through its application in an information retrieval system. A performance comparison between this system and a traditional keyword-based approach, using precision, recall, and F1 measure, reveals that the CoVPO-based query expansion search system outperforms the traditional method, achieving higher F1 scores, recall, and precision across various search queries and datasets.

In future, we plan to enhance the current CoVPO ontology by integrating more SARS-CoV-2 datasets available online and publishing CoVPO using a triplestore database as a SPARQL endpoint. This will improve its capacity to handle larger datasets. Furthermore, we aim to expand CoVPO by incorporating additional use cases.

## Supporting information

S1 File SARS-CoV-2 protein ontology. This file provides the constructed ontology used in the study.
(OWL)
